# Positive Psychological Well-Being in Early Palliative Care: A Narrative Review of the Roles of Hope, Gratitude, and Death Acceptance

**DOI:** 10.3390/curroncol31020049

**Published:** 2024-01-24

**Authors:** Elena Bandieri, Eleonora Borelli, Sarah Bigi, Claudia Mucciarini, Fabio Gilioli, Umberto Ferrari, Sonia Eliardo, Mario Luppi, Leonardo Potenza

**Affiliations:** 1 Oncology and Palliative Care Units, Civil Hospital Carpi, Unità Sanitaria Locale (USL), 41012 Carpi, Italy; e.bandieri@ausl.mo.it (E.B.); c.mucciarini@ausl.mo.it (C.M.); f.gilioli@ausl.mo.it (F.G.); u.ferrari@ausl.mo.it (U.F.); s.eliardo@ausl.mo.it (S.E.); 2 Department of Medical and Surgical Sciences, University of Modena and Reggio Emilia, 41124 Modena, Italy; mario.luppi@unimore.it (M.L.); leonardo.potenza@unimore.it (L.P.); 3 Department of Linguistic Sciences and Foreign Literatures, Catholic University of the Sacred Heart, 20123 Milan, Italy; sarah.bigi@unicatt.it; 4 Hematology Unit and Chair, Azienda Ospedaliera Universitaria di Modena, 41124 Modena, Italy

**Keywords:** early palliative care, positive psychology, advanced cancer, hope, gratitude, death acceptance

## Abstract

In the advanced cancer setting, low psychological functioning is a common symptom and its deleterious impact on health outcomes is well established. Yet, the beneficial role of positive psychological well-being (PPWB) on several clinical conditions has been demonstrated. Early palliative care (EPC) is a recent value-based model consisting of the early integration of palliative care into standard care for solid tumors and hematologic malignancies. While the late palliative care primary offers short-term interventions, predominantly pharmacological in nature and limited to physical symptom reduction, EPC has the potential to act over a longer term, enabling specific interventions aimed at promoting PPWB. This narrative review examines nine English studies retrieved from MEDLINE/PubMed, published up to October 2023, focusing on EPC and three dimensions of PPWB: hope, gratitude, and death acceptance. These dimensions consistently emerge in our clinical experience within the EPC setting for advanced cancer patients and appear to contribute to its clinical efficacy. The choice of a narrative review reflects the novelty of the topic, the limited existing research, and the need to incorporate a variety of methodological approaches for a comprehensive exploration.

## 1. Introduction

A growing body of evidence highlights an intriguing interconnection between psychological health and physical health, suggesting how the former can impact the latter for better or for worse. Historically, most of the research focused on understanding the impact of negative psychological health, with comparatively limited exploration into the effects of positive psychological health [1,2,3]. However, the effects of psychological well-being and psychological ill-being are not merely, opposites and the presence of one does not necessarily imply the absence of the other [4]. As a result, scholars have recently delved into the specific exploration of the benefits of positive psychological well-being (PPWB) on health [5,6,7,8] and reported that these surpass the adverse effects of negative psychology [3,5,6,7,8,9,10,11].

In the literature, the concept of PPWB is defined as a comprehensive umbrella referring to cognitive and emotional constructs employed by individuals who assess their lives favorably and thrive [3,5,12]. It may include several factors, namely optimism, gratitude, perseverance, acceptance, satisfaction, life purpose, and happiness. However, the cognitive and emotional constructs associated with PPWB may differ based on the specific theoretical framework under consideration. Among the main frameworks, the hedonic model of well-being pertains to the pursuit of happiness and pleasure [13] and includes the constructs of life satisfaction and positive and negative effects [14]. The eudemonic model of well-being refers to the realization of one’s potential and the pursuit of a meaningful life [15] and includes the constructs of self-acceptance, positive relations, autonomy, environmental mastery, purpose in life, and personal growth [16]. The social model of well-being encompasses a more extensive social viewpoint [17] and refers to the extent to which individuals are adeptly addressing social challenges and operating proficiently within their social environment [18]. It includes the constructs of social integration, social contribution, social coherence, social actualization, and social acceptance. Several other potential PPWB constructs (e.g., optimism, vitality, humor) defy straightforward classification within just one framework and, despite encompassing aspects of more models, do not neatly fit into either one [19]. Moreover, the application of PPWB may be hindered by a distinct categorization within one theoretical framework rather than another [18].

While PPWB has primarily been explored in non-clinical samples [1], there is some evidence indicating its positive influence on clinical outcomes and the usefulness of dedicated interventions in various illnesses [1,5,6,9,12,20,21,22]. Studies on PPWB in the setting of advanced cancer are scarce [1,2,23,24,25,26]. However, incorporating its evaluation and interventions into clinical routines, which are currently predominantly concentrated on assessing psychological ill-being, could be crucial for attaining comprehensive well-being along the disease trajectory. This approach may empower patients not only to endure but also to flourish, irrespective of whether they are dealing with early-stage or advanced cancer [27].

Nevertheless, it is reasonable to speculate that the notion of PPWB may vary in the context of a terminal illness as opposed to a state of health or a non-terminal illness. Confronting a dire diagnosis changes the patient’s outlook and reshapes their priorities, and what typically fosters well-being might undergo alterations [28]. Thus, in order to be correctly assessed and addressed, the concept of PPWB should be reframed in clinical settings, like oncology and hematology, in which the physician’s focus may need to shift from cure to end-of-life quality of life.

**Figure 1:**
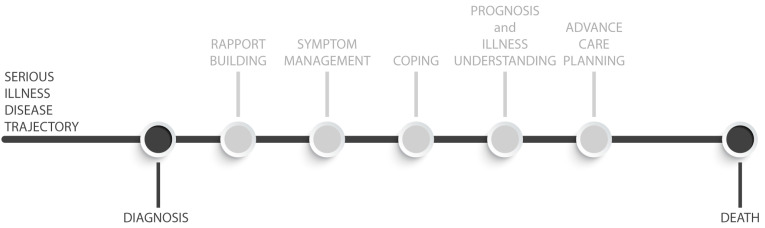
EPC complex intervention.

Recently, a care model has emerged in the oncology and onco-hematology settings, that, despite not being inherently integrated into the framework of the PPWB, underscores its fundamental principles. This model is known as Early Palliative Care (EPC). EPC is defined as the timely integration of palliative care into standard care, usually within 8 weeks of diagnosing incurable cancer, for patients with a prognosis of 6–24 months and their caregivers [29,30,31,32], in contrast to the conventional model of palliative care, which is typically administered in the last months of life [33,34,35] (Figure 1). In conventional palliative care, clinicians primarily offer consultations to hospitalized patients facing serious illnesses in the final stages of life, requiring acute symptom management, advanced care planning, and referral to hospice services. Conversely, in EPC, clinicians have the opportunity to establish ongoing connections with patients and their families. This broadens their scope from mere symptom management to actively assisting patients in leading fulfilling lives by adapting and effectively coping with the challenges of serious illness, focusing on assessing and addressing their physical, emotional, social, and spiritual needs. EPC has demonstrated positive effects on various aspects of patient care, i.e., quality of life and mood [36,37,38,39,40,41], awareness of prognosis [42,43], and symptom management [35]. Additionally, it has shown the potential to lower the risk of severe pain [29,38], decrease the use of chemotherapy near the end of life, reduce the aggressiveness of therapy, increase hospice care enrollments [35,36,37,44], and even improve survival rates [37,45]. Moreover, the benefits of EPC extend to caregivers in randomized clinical trials involving cancer patients [46]. Caregivers reported lower levels of depression and burden [39,43,47], improvements in coping strategies [39], and greater satisfaction with the provided care [48]. More recent reports have also suggested positive outcomes of EPC for patients with hematologic diseases [39,49,50,51,52,53]. EPC offers an ideal environment for either assessing or addressing PPWB through the incorporation of dedicated interventions, for instance, fostering patients’ reflection on and expression of gratitude for positive aspects in their lives, empowering them, optimizing their personal coping strategies, facilitating connections with loved ones and with a supportive social network, and supporting them to engage in activities they find enjoyable and meaningful.

The aim of the present work is to review three constructs, namely hope, gratitude, and death acceptance, which may be included among the indicators of PPWB in the setting of advanced cancer, beyond others. Drawing from our clinical experience and the literature, these constructs have been found to emerge in the EPC setting, contribute to its clinical effect, and may represent some of the means through which EPC exerts an enhancing effect on the PPWB of both patients and caregivers. The role of hope in PPWB has been extensively investigated, demonstrating a positive correlation with resilience, a mediating role in various relationships, and significance in diverse contexts, including palliative care. Gratitude, although less explored in the palliative care setting, has been observed in patients and caregivers undergoing EPC. Despite being unsolicited, gratitude can serve as a valuable resource in EPC interventions. Therefore, assessing gratitude and implementing gratitude-based interventions could prove beneficial in the EPC context. Conversations about death are considered a desirable practice for patients and caregivers in interactions with physicians, and their acceptance appears to contribute to alleviating the fear of the future.

## 2. Materials and Methods

This narrative review was conceived as an overview of the scientific literature, incorporating a critical analysis of the gathered evidence. General recommendations aimed at enhancing the quality of narrative reviews in the medical area were taken into account [54].

### 2.1. Selection Criteria and Search Source

Inclusion and exclusion criteria were established for eligible studies. Inclusion criteria encompassed any studies written in English and published until October 2023 on EPC in adult patients with advanced cancer or their caregivers, specifically addressing the themes of hope, gratitude, or death acceptance in their titles or abstracts. All types of articles were considered eligible for inclusion. The only exclusion criterion applied was specific to the pediatric population within the sample.

In this review, simultaneous care and supportive care are considered synonyms for EPC; caregiver refers to any relatives, friends, or partners who provide uncompensated aid to a person with a serious or chronic life-threatening illness.

After consulting with a librarian specializing in health sciences, the search was conducted in the MEDLINE/PubMed electronic databases.

### 2.2. Search Terms

The search string used was (Cancer [Title/Abstract] OR Oncology [Title/Abstract] OR Tumor [Title/Abstract]) AND (“Simultaneous palliative care”[Title/Abstract] OR “Early palliative care”[Title/Abstract] OR “Supportive palliative care”[Title/Abstract]) AND (Hope[Title/Abstract] OR Gratitude[Title/Abstract] OR “Acceptance of death”[Title/Abstract] OR “Death acceptance”[Title/Abstract]).

## 3. Results and Discussion

**Figure 2:**
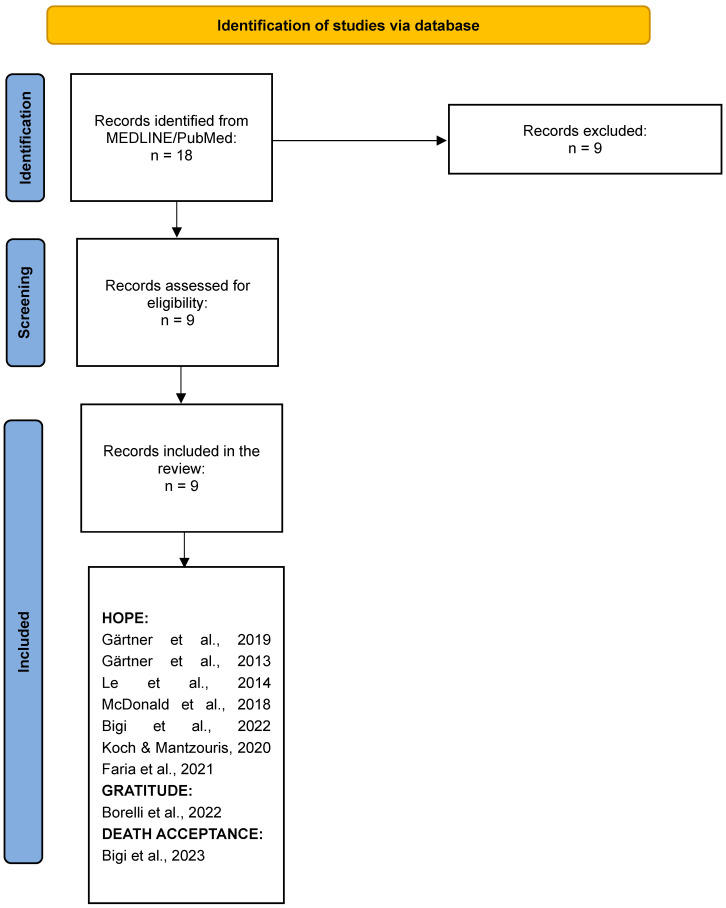
Flow diagram of study selection [55,56,57,58,59,60,61,62,63].

A total of 18 studies were identified from the electronic search on MEDLINE/PubMed. Four studies were excluded because they included a pediatric population; three studies were excluded because they mentioned that the word “hope” referred to either treatments or breakthrough interventions or the context of compassionate use programs; one study was excluded because it mentioned hope in the abstract but did not develop the topic in the main text; one study was excluded because it discussed “hope” by reporting the results of a study already present in the retrieved list. Nine studies were included in the review. Out of the nine studies retrieved on EPC, seven addressed the theme of hope, one focused on gratitude, and one explored the theme of death acceptance (Figure 2).

### 3.1. Hope

Hope emerged as the most frequently cited of the three constructs in studies within the EPC setting. Specifically, we identified reference to the concept of hope in the context of EPC in 7 out of 9 retrieved studies, although without explicit mention of PPWB.

In two of these studies, Gärtner and colleagues [55,56] provided a summary of available information on the early integration of both general/primary palliative care and specialized palliative care in the advanced cancer setting. They offered practical recommendations derived from their clinical experience with the model in Germany, addressing the theme of hope when discussing the characteristics that medical-patient communication should embody.

Three studies explore the theme of hope through qualitative analyses of verbal reports, and only one of them specifically focuses on the construct of hope. In 2014, Le and colleagues [57] conducted a qualitative analysis based on data obtained from three focus groups and six individual interviews involving 28 healthcare professionals engaged in managing lung cancer in Melbourne, Australia. The objective was to explore their attitudes toward palliative care, specifically its early integration. The study consistently brought forth the theme of hope, with healthcare professionals expressing concerns about potentially diminishing it when proposing the introduction of palliative care. Later on, in 2018, McDonald and colleagues [58] interviewed 23 caregivers who had participated in a cluster-randomized trial of EPC in Toronto, Canada. They qualitatively analyzed the caregivers’ reports to define and compare the construct of quality of life for those who received or did not receive the EPC intervention. In this instance, the theme of hope emerged prominently in the reports of caregivers who received EPC, especially in discussions about mortality. Bigi and colleagues [59] conducted a content and lexicographic analysis of responses to open-ended questionnaires administered to 36 bereaved caregivers of patients who received EPC at two Italian cancer centers. In this work, the aim was to explore their perceptions of hope.

Koch and Mantzouris [60] and Faria and colleagues [61] described clinical cases where a failure to initiate EPC resulted in patients seeking aggressive treatments and eventually dying without being informed of their true life expectancy. In these cases, the contrasting meanings that hope may assume for stakeholders in a terminal illness context (healing vs. achieving optimal quality of life) are explored, and depending on the attributed meaning, the outcome of the situation can be either unfavorable or desirable.

In general, hope is considered to play a crucial role in improving health by influencing individuals’ goal-setting, self-confidence and motivation, performances in attaining goals, and overall well-being, thereby impacting health-related behaviors and outcomes [64]. The experience of falling ill, when faced with hope, leads individuals to channel their energy towards the expectation of health restoration and spiritual well-being, thereby contributing to improving health outcomes [65,66].

In the oncological context, hope seems to be a dynamic concept that has been associated with coping, resilience, and improved quality of life for both patients and caregivers. They considered it an important resource to deal with the complexities of the diagnosis, playing a significant role in motivating them through disorienting grief towards an integrated sense of self [28,67,68,69,70]. The significance of hope in the context of advanced cancer is further emphasized by its association with decreased passivity, better quality of life, and less pain and anxiety in patients [67]. A Living with Hope Program has been developed to foster hope in advanced cancer patients and their caregivers, once its benefits have been acknowledged [71]. In the caregiving context, hope has been identified as a psychosocial and spiritual resource used by caregivers to manage and deal with the caregiver experience [71] and a determinant for psychiatric morbidity [72].

In the context of advanced cancer, it may seem challenging to envision a chance of maintaining realistic hope. Gärtner and colleagues [56], Le and colleagues [57], and Odejide and colleagues [73,74] reported clinician concern about taking away hope emerging as a barrier hindering oncologists and hematologists from recommending EPC to cancer patients. The clinicians expressed apprehensions regarding the potential erosion of hope in patients and their caregivers stemming from the prevailing stigma associating palliative care as an intervention primarily implemented in end-of-life scenarios [75].

Yet, hope may be crucial for both patients’ and caregivers’ psychological and emotional well-being. Aligned with Alfred Adler’s proposition that humans ‘cannot think, feel, will, or act without the perception of a goal’ [76], physicians recognize that communicating hope is an integral facet of patient care. Cancer care guidelines formally acknowledged the provision of hope as an integral component of palliative care [77].

Gärtner and colleagues [56] enumerated, within the framework of the ten fundamental principles for cancer pain management, the imperative to identify factors—beyond the strictly physical or disease-associated elements—contributing to patient total pain [78]. They spotlighted existential suffering linked to the loss of hope as one of these influential factors. In accordance with the Authors, oncologists should proactively seek information during initial visits regarding who would and who would not be involved in the information and decision-making processes, also regarding end-of-life care, based on studies showing that a significant proportion of patients express a preference for involvement [79]. Within this context, the concept of hope assumes paramount significance for physicians. Healthcare professionals frequently articulate that their ‘fear of diminishing hope’ constitutes a central obstacle to participating in advance care planning and conversations concerning end-of-life care [79,80].

It is crucial to underscore that, particularly for patients with advanced cancer and other incurable and life-threatening conditions, physicians should refrain from conveying a unidimensional concept of hope solely grounded in the prospect of cure or disease ‘control’. Instead, the provision of hope hinges on considerations of dignity, comfort, closure, and growth at the end of life.

To effectively approach this responsibility, physicians necessitate empathy, i.e., the willingness to perceive, identify, and acknowledge the existential dimension of patients’ suffering, which is imperative for their capacity to provide hope [81].

McDonald and colleagues [48] investigated the quality of life construct for caregivers of advanced cancer patients on EPC from their own perspective and compared it with that of caregivers of advanced cancer patients not on EPC. When discussing the challenge of confronting mortality, hope emerged as a significant theme contributing to the overall quality of life. Caregivers in the EPC group demonstrated the ability to balance hope with realism. This balance was manifested in two ways: for some, it involved maintaining hope that the illness may regress or be cured while acknowledging the improbability of such an occurrence, while for others, it entailed redefining the object of hope and shifting it from the illness outcome to the overall quality of life.

These two forms of hope in EPC were also found by Bigi and colleagues [59] who explored the perception of hope among caregivers of deceased patients with advanced cancer in EPC. They utilize a lexicographic analysis of responses to an open-ended questionnaire. The study reveals hope as a dynamic concept, consistently redefined throughout the course of the illness, transcending the dichotomy of life and death. Instead, it is shaped by elements that characterize the continuum of quality of life from “healthy” to “suffering”. Notably, hope is intertwined with the concepts of resilience, expectation, and desire. Caregivers attributed the foundation of hope to the truth and trust provided by the EPC medical team. They established a connection between hope, the EPC intervention, and the meaningful relationships cultivated with the palliative care specialist. The primary outcome of the analysis centers around the notion of “realistic hope,” signifying an expectation of positive outcomes firmly grounded in realistic expectations.

Realistic hope has been defined by Gärtner and colleagues [56] as “double awareness”. The authors described in their study the role of healthcare professionals, regardless of their discipline, in delivering “general” or “primary” palliative care. Since one of the primary concerns of non-palliative care physicians in providing EPC is the fear of diminishing the patient’s hope, the authors clarify that understanding what is meant by “hope” in the context of oncological illness is essential. According to the National Comprehensive Cancer Center Network (NCCN), “hope” in the context of incurable and life-threatening cancer involves setting realistic goals, such as being free of pain or not feeling alone [77]. Honest, empathetic, and truthful disclosure is associated with hope, even when delivering unfavorable news or providing a poor prognosis. For these authors, hope should be understood as enabling the patient and caregiver to prepare for a realistic course of the illness while still supporting the improbable expectation of improvement. This approach allows patients to “hope for the best but be prepared for other outcomes”. Indeed, in the EPC setting, clinicians make hope a multidimensional domain by typically providing coping support, exploring and expanding the “land” of hope, and redirecting it. This is provided along the entire trajectory of cancer, along with supportive counseling about the illness trajectory and discussing behavioral strategies [82,83,84].

We also found two case reports that are exemplifications of the need for hope in terminally ill patients. Koch and Mantzouris [60] reported the story of a young mother diagnosed with stage IV cancer who opted for aggressive treatment, with her healthcare team failing to communicate to her or her family the imminent death. While detailing the case, the authors recognized that conveying the prognosis and the approach to end of life does not take away hope; instead, it reframes it as an attainable quality of life. Faria and colleagues [61] detailed the case of a 53-year-old male diagnosed with stage IV breast cancer who experienced unmanaged pain resulting from physical, emotional, social, and existential distress. The authors underscore how, despite being aware of having an incurable disease, the patient retained optimistic expectations concerning potential treatment options. They stress how his unrealistic hope for a positive outcome, coupled with his awareness of the absence of a permanent cure, led to ambivalent thoughts that ultimately contributed to his existential suffering, and proposed that EPC, which had not been offered to the patient, could have improved his quality of life through the course of his disease.

### 3.2. Gratitude

Only one paper mentioned the construct of gratitude in the context of EPC. Borelli and colleagues [62] explored the manifestation and expressions of gratitude in 133 patients with advanced cancer receiving EPC and 118 caregivers of both living and deceased patients who were or have been on EPC from two Italian outpatient EPC units. The authors conducted a qualitative and quantitative analysis of transcripts of open-ended questionnaires that detailed their clinical experiences with the intervention. The authors found that gratitude predominantly stemmed from effective management of physical symptoms, emotional support provided by the EPC team, a more positive attitude towards death, enhanced information, human connection, and the familiar environment of the EPC units and that it may be considered as an indirect, secondary outcome of EPC.

Gratitude plays a pivotal role in improving health among healthy individuals, influencing various aspects of well-being, and contributing to positive health outcomes. Research has consistently demonstrated the significant impact of gratitude on psychological, emotional, and physical health, highlighting its multifaceted role in promoting overall well-being. It has been associated with enhanced subjective well-being, including increased life satisfaction, happiness, and positive affect [85]. Wood and colleagues [85] conducted a review of the theoretical integration of gratitude and well-being, emphasizing the positive relationship between gratitude and subjective well-being. Furthermore, gratitude has been linked to reduced stress and depression, with longitudinal studies demonstrating its role in the development of social support and resilience [86]. The connection between gratitude and psychological well-being, as well as positive social relationships, has been highlighted in the literature. Conversely, the research linking gratitude to physical well-being is somewhat limited, despite the potential profound impact that such a connection could have on a cancer population facing a life-threatening diagnosis. If the connection between gratitude and physical well-being were to be confirmed, healthcare professionals should adopt care models that have the potential to foster it [62].

The exploration of gratitude’s role in the EPC setting has only recently gained attention. This interest arises from the observation that gratitude has been specifically associated with psychological dimensions that palliative assessment assesses and addresses [62]. In patients, gratitude seems to arise from close bonds with family and friends and to be positively associated with health status, quality of life, appreciation of life, and the post-traumatic growth dimension and negatively associated with psychological distress [87]. In caregivers, gratitude seems to arise from what is acknowledged as the pillars of the doctor-patient relationship in EPC, such as humanity, professionalism, and emotional support, along with a *holistic* approach to illness [88].

### 3.3. Death Acceptance

Only Bigi and colleagues [63] mentioned the construct of death acceptance in the context of EPC. The authors analyzed transcripts of open-ended questionnaires administered to 130 patients with advanced cancer receiving EPC and 115 caregivers of both living and deceased patients who were or have been on EPC from two Italian outpatient EPC units by qualitatively and quantitatively comparing them with texts collected from an Italian forum, which included instances of web-mediated interactions between patients and their caregivers, presumably not on EPC. The objective was to elucidate how EPC may influence the perception of death. The findings indicated that discussing death is not taboo for both patients on EPC and their caregivers, and it is not necessarily associated with negative connotations. In fact, both in the transcripts of the open-ended questionnaires and in the Italian forum, words explicitly referring to the experience of death were frequently combined with words expressing its rationalization or acceptance. However, while in the EPC transcripts, positive representations of death were portrayed as an actual and positive experience of the end of life, in the forum, they were depicted as an unattainable wish or desire. The realistic description of patients’ prognoses, with the support of a multidisciplinary EPC team, could contribute to helping patients with cancer accept their death and prepare for it.

## 4. Conclusions

Multidisciplinary research should be encouraged in order to analyze the PPWB concept in the context of EPC in the advanced cancer setting. Anticipation of palliative interventions allowing long-term rapport building may favor the re-framing of some constructs such as hope, gratitude, and death. EPC appears to be an ideal clinical model for building rapport and implementing effective communication between physicians and patients/caregivers about illness understanding, realistic expectations, acceptance, expansion of hopes, goals of care, planning the future, and death. PPWB and a positive experience of death may indeed be favored in an EPC setting. Patient-centered communication skills should be implemented to overcome communication deficiencies, as it is through medical-patient communication that concepts of hope, gratitude, and death acceptance can be nurtured. Therefore, any intervention on these three constructs cannot be inseparable from the patient-clinician relationship. The roles of education and system intervention should be a priority to address patient-clinician communication issues and to improve the value of medical care for advanced cancer patients [89].

The limitation of this study is its adherence to the results obtained from the search conducted on MEDLINE/PubMed, without extending the inquiry to articles from the reference lists of the retrieved papers. As a consequence, only one paper was found for two out of the three constructs investigated. The rationale behind this methodological choice is that the constructs mentioned in the works cited in the reference lists were not analyzed from the perspective of EPC. However, the limited number of retrieved works reveals a gray area in the study of PPWB in the context of EPC, which warrants further investigation.

## References

[1] Chakhssi F., Kraiss J.T., Sommers-Spijkerman M., Bohlmeijer E.T. (2018). The Effect of Positive Psychology Interventions on Well-Being and Distress in Clinical Samples with Psychiatric or Somatic Disorders: A Systematic Review and Meta-Analysis. BMC Psychiatry.

[2] Amonoo H.L., Lam J.A., Daskalakis E., Deary E.C., Celano C., Onyeaka H.K., Newcomb R., Barata A., Horick N., Cutler C. (2023). Positive Psychological Well-Being in Hematopoietic Stem Cell Transplantation Survivors. Transplant. Cell. Ther..

[3] Boehm J.K., Kubzansky L.D. (2012). The Heart’s Content: The Association between Positive Psychological Well-Being and Cardiovascular Health. Psychol. Bull..

[4] Ryff C.D., Dienberg Love G., Urry H.L., Muller D., Rosenkranz M.A., Friedman E.M., Davidson R.J., Singer B. (2006). Psychological Well-Being and Ill-Being: Do They Have Distinct or Mirrored Biological Correlates?. Psychother. Psychosom..

[5] Kubzansky L.D., Huffman J.C., Boehm J.K., Hernandez R., Kim E.S., Koga H.K., Feig E.H., Lloyd-Jones D.M., Seligman M.E.P., Labarthe D.R. (2018). Positive Psychological Well-Being and Cardiovascular Disease. J. Am. Coll. Cardiol..

[6] Levine G.N., Cohen B.E., Commodore-Mensah Y., Fleury J., Huffman J.C., Khalid U., Labarthe D.R., Lavretsky H., Michos E.D., Spatz E.S. (2021). Psychological Health, Well-Being, and the Mind-Heart-Body Connection: A Scientific Statement From the American Heart Association. Circulation.

[7] Chida Y., Steptoe A. (2008). Positive Psychological Well-Being and Mortality: A Quantitative Review of Prospective Observational Studies. Psychosom. Med..

[8] Martín-María N., Miret M., Caballero F.F., Rico-Uribe L.A., Steptoe A., Chatterji S., Ayuso-Mateos J.L. (2017). The Impact of Subjective Well-Being on Mortality: A Meta-Analysis of Longitudinal Studies in the General Population. Psychosom. Med..

[9] Huffman J.C., Feig E.H., Zambrano J., Celano C.M. (2023). Positive Psychology Interventions in Medical Populations: Critical Issues in Intervention Development, Testing, and Implementation. Affect. Sci..

[10] Diener E., Chan M.Y. (2011). Happy People Live Longer: Subjective Well-Being Contributes to Health and Longevity: Health Benefits of Happiness. Appl. Psychol. Health Well-Being.

[11] Pressman S.D., Cohen S. (2005). Does Positive Affect Influence Health?. Psychol. Bull..

[12] Boehm J.K., Peterson C., Kivimaki M., Kubzansky L. (2011). A Prospective Study of Positive Psychological Well-Being and Coronary Heart Disease. Health Psychol..

[13] Keyes C.L.M., Annas J. (2009). Feeling Good and Functioning Well: Distinctive Concepts in Ancient Philosophy and Contemporary Science. J. Posit. Psychol..

[14] Diener E., Suh E.M., Lucas R.E., Smith H.L. (1999). Subjective Well-Being: Three Decades of Progress. Psychol. Bull..

[15] Waterman A.S. (2007). Doing Well: The Relationship of Identity Status to Three Conceptions of Well-Being. Identity.

[16] Ryff C.D. (1989). Happiness Is Everything, or Is It? Explorations on the Meaning of Psychological Well-Being. J. Pers. Soc. Psychol..

[17] Keyes C.L.M. (1998). Social Well-Being. Soc. Psychol. Q..

[18] Gallagher M.W., Lopez S.J., Preacher K.J. (2009). The Hierarchical Structure of Well-Being. J. Pers..

[19] Gallagher M.W., Lopez S.J. (2009). Positive Expectancies and Mental Health: Identifying the Unique Contributions of Hope and Optimism. J. Posit. Psychol..

[20] Huffman J.C., DuBois C.M., Millstein R.A., Celano C.M., Wexler D. (2015). Positive Psychological Interventions for Patients with Type 2 Diabetes: Rationale, Theoretical Model, and Intervention Development. J. Diabetes Res..

[21] Huffman J.C., Millstein R.A., Mastromauro C.A., Moore S.V., Celano C.M., Bedoya C.A., Suarez L., Boehm J.K., Januzzi J.L. (2016). A Positive Psychology Intervention for Patients with an Acute Coronary Syndrome: Treatment Development and Proof-of-Concept Trial. J. Happiness Stud..

[22] Huffman J.C., Legler S.R., Boehm J.K. (2017). Positive Psychological Well-Being and Health in Patients with Heart Disease: A Brief Review. Future Cardiol..

[23] Chen J., Xiao H., Chen Y., Sun H., Chen S., Zheng J. (2020). Effect of Reminiscence Therapy Based on Positive Psychology Theory (RTBPPT) on the Positive Feelings of the Spousal Caregivers of Elderly Patients with Advanced Cancer in China. Eur. J. Cancer Care.

[24] Tang S.T., Lin K.-C., Chen J.-S., Chang W.-C., Hsieh C.-H., Chou W.-C. (2015). Threatened with Death but Growing: Changes in and Determinants of Posttraumatic Growth over the Dying Process for Taiwanese Terminally Ill Cancer Patients. Psychooncology.

[25] Amonoo H.L., Daskalakis E., Deary E.C., Celano C.M., Ghanime P.M., Healy B.C., Cutler C., Pirl W.F., Park E.R., Gudenkauf L.M. (2023). Feasibility of a Positive Psychology Intervention (PATH) in Allogeneic Hematopoietic Stem Cell Transplantation Survivors: Randomized Pilot Trial Design and Methods. Contemp. Clin. Trials.

[26] Casellas-Grau A., Font A., Vives J. (2014). Positive Psychology Interventions in Breast Cancer. A Systematic Review: Positive Interventions in Breast Cancer. Psychooncology.

[27] Amonoo H.L., El-Jawahri A., Deary E.C., Traeger L.N., Cutler C.S., Antin J.A., Huffman J.C., Lee S.J. (2022). Yin and Yang of Psychological Health in the Cancer Experience: Does Positive Psychology Have a Role?. J. Clin. Oncol..

[28] Currin-McCulloch J., Walsh C., Gulbas L., Trevino K., Pomeroy E., Jones B. (2021). Contingent Hope Theory: The Developmental Exploration of Hope and Identity Reconciliation among Young Adults with Advanced Cancers. Palliat. Support. Care.

[29] Temel J.S., Greer J.A., Muzikansky A., Gallagher E.R., Admane S., Jackson V.A., Dahlin C.M., Blinderman C.D., Jacobsen J., Pirl W.F. (2010). Early Palliative Care for Patients with Metastatic Non–Small-Cell Lung Cancer. N. Engl. J. Med..

[30] Rodin G., Malfitano C., Rydall A., Schimmer A., Marmar C.M., Mah K., Lo C., Nissim R., Zimmermann C. (2020). Emotion And Symptom-Focused Engagement (EASE): A Randomized Phase II Trial of an Integrated Psychological and Palliative Care Intervention for Patients with Acute Leukemia. Support. Care Cancer.

[31] Potenza L., Borelli E., Bigi S., Giusti D., Longo G., Odejide O., Porro C.A., Zimmermann C., Efficace F., Bruera E. (2022). Early Palliative Care in Acute Myeloid Leukemia. Cancers.

[32] Ferrell B.R., Temel J.S., Temin S., Alesi E.R., Balboni T.A., Basch E.M., Firn J.I., Paice J.A., Peppercorn J.M., Phillips T. (2017). Integration of Palliative Care Into Standard Oncology Care: American Society of Clinical Oncology Clinical Practice Guideline Update. J. Clin. Oncol..

[33] Ferrell B.R. (2005). Late Referrals to Palliative Care. J. Clin. Oncol..

[34] Borelli E., Bigi S., Potenza L., Gilioli F., Efficace F., Porro C.A., Luppi M., Bandieri E. (2023). Caregiver’s Quality of Life in Advanced Cancer: Validation of the Construct in a Real-Life Setting of Early Palliative Care. Front. Oncol..

[35] Bandieri E., Banchelli F., Artioli F., Mucciarini C., Razzini G., Cruciani M., Potenza L., D’Amico R., Efficace F., Bruera E. (2019). Early versus Delayed Palliative/Supportive Care in Advanced Cancer: An Observational Study. BMJ Support. Palliat. Care.

[36] Maltoni M., Scarpi E., Dall’Agata M., Zagonel V., Bertè R., Ferrari D., Broglia C.M., Bortolussi R., Trentin L., Valgiusti M. (2016). Systematic versus On-Demand Early Palliative Care: Results from a Multicentre, Randomised Clinical Trial. Eur. J. Cancer.

[37] Hannon B., Swami N., Pope A., Leighl N., Rodin G., Krzyzanowska M., Zimmermann C. (2016). Early Palliative Care and Its Role in Oncology: A Qualitative Study. Oncologist.

[38] Bandieri E., Sichetti D., Romero M., Fanizza C., Belfiglio M., Buonaccorso L., Artioli F., Campione F., Tognoni G., Luppi M. (2012). Impact of Early Access to a Palliative/Supportive Care Intervention on Pain Management in Patients with Cancer. Ann. Oncol..

[39] El-Jawahri A., LeBlanc T., VanDusen H., Traeger L., Greer J.A., Pirl W.F., Jackson V.A., Telles J., Rhodes A., Spitzer T.R. (2016). Effect of Inpatient Palliative Care on Quality of Life 2 Weeks After Hematopoietic Stem Cell Transplantation: A Randomized Clinical Trial. JAMA.

[40] Vanbutsele G., Pardon K., Van Belle S., Surmont V., De Laat M., Colman R., Eecloo K., Cocquyt V., Geboes K., Deliens L. (2018). Effect of Early and Systematic Integration of Palliative Care in Patients with Advanced Cancer: A Randomised Controlled Trial. Lancet Oncol..

[41] Zimmermann C., Swami N., Krzyzanowska M., Hannon B., Leighl N., Oza A., Moore M., Rydall A., Rodin G., Tannock I. (2014). Early Palliative Care for Patients with Advanced Cancer: A Cluster-Randomised Controlled Trial. Lancet.

[42] Temel J.S., Greer J.A., Admane S., Gallagher E.R., Jackson V.A., Lynch T.J., Lennes I.T., Dahlin C.M., Pirl W.F. (2011). Longitudinal Perceptions of Prognosis and Goals of Therapy in Patients With Metastatic Non–Small-Cell Lung Cancer: Results of a Randomized Study of Early Palliative Care. J. Clin. Oncol..

[43] Temel J.S., Greer J.A., El-Jawahri A., Pirl W.F., Park E.R., Jackson V.A., Back A.L., Kamdar M., Jacobsen J., Chittenden E.H. (2017). Effects of Early Integrated Palliative Care in Patients With Lung and GI Cancer: A Randomized Clinical Trial. J. Clin. Oncol..

[44] Greer J.A., Pirl W.F., Jackson V.A., Muzikansky A., Lennes I.T., Heist R.S., Gallagher E.R., Temel J.S. (2012). Effect of Early Palliative Care on Chemotherapy Use and End-of-Life Care in Patients With Metastatic Non–Small-Cell Lung Cancer. J. Clin. Oncol..

[45] Bakitas M.A., Tosteson T.D., Li Z., Lyons K.D., Hull J.G., Li Z., Dionne-Odom J.N., Frost J., Dragnev K.H., Hegel M.T. (2015). Early Versus Delayed Initiation of Concurrent Palliative Oncology Care: Patient Outcomes in the ENABLE III Randomized Controlled Trial. J. Clin. Oncol..

[46] Alam S., Hannon B., Zimmermann C. (2020). Palliative Care for Family Caregivers. J. Clin. Oncol..

[47] Dionne-Odom J.N., Azuero A., Lyons K.D., Hull J.G., Tosteson T., Li Z., Li Z., Frost J., Dragnev K.H., Akyar I. (2015). Benefits of Early Versus Delayed Palliative Care to Informal Family Caregivers of Patients With Advanced Cancer: Outcomes from the ENABLE III Randomized Controlled Trial. J. Clin. Oncol..

[48] McDonald J., Swami N., Hannon B., Lo C., Pope A., Oza A., Leighl N., Krzyzanowska M.K., Rodin G., Le L.W. (2017). Impact of Early Palliative Care on Caregivers of Patients with Advanced Cancer: Cluster Randomised Trial. Ann. Oncol..

[49] El-Jawahri A., Traeger L., Greer J.A., VanDusen H., Fishman S.R., LeBlanc T.W., Pirl W.F., Jackson V.A., Telles J., Rhodes A. (2017). Effect of Inpatient Palliative Care During Hematopoietic Stem-Cell Transplant on Psychological Distress 6 Months After Transplant: Results of a Randomized Clinical Trial. J. Clin. Oncol..

[50] El-Jawahri A., LeBlanc T.W., Kavanaugh A., Webb J.A., Jackson V.A., Campbell T.C., O’Connor N., Luger S.M., Gafford E., Gustin J. (2021). Effectiveness of Integrated Palliative and Oncology Care for Patients With Acute Myeloid Leukemia: A Randomized Clinical Trial. JAMA Oncol..

[51] El-Jawahri A., Nelson A.M., Gray T.F., Lee S.J., LeBlanc T.W. (2020). Palliative and End-of-Life Care for Patients With Hematologic Malignancies. J. Clin. Oncol..

[52] Giusti D., Colaci E., Pioli V., Banchelli F., Maccaferri M., Leonardi G., Marasca R., Morselli M., Forghieri F., Bettelli F. (2023). Early Palliative Care versus Usual Haematological Care in Multiple Myeloma: Retrospective Cohort Study. BMJ Support. Palliat. Care.

[53] Potenza L., Scaravaglio M., Fortuna D., Giusti D., Colaci E., Pioli V., Morselli M., Forghieri F., Bettelli F., Messerotti A. (2021). Early Palliative/Supportive Care in Acute Myeloid Leukaemia Allows Low Aggression End-of-Life Interventions: Observational Outpatient Study. BMJ Support. Palliat. Care.

[54] Ferrari R. (2015). Writing Narrative Style Literature Reviews. Med. Writ..

[55] Gärtner J., Daun M., Wolf J., von Bergwelt-Baildon M., Hallek M. (2019). Early Palliative Care: Pro, but Please Be Precise!. Oncol. Res. Treat..

[56] Gaertner J., Weingärtner V., Wolf J., Voltz R. (2013). Early Palliative Care for Patients with Advanced Cancer: How to Make It Work?. Curr. Opin. Oncol..

[57] Le B.H.C., Mileshkin L., Doan K., Saward D., Spruyt O., Yoong J., Gunawardana D., Conron M., Philip J. (2014). Acceptability of Early Integration of Palliative Care in Patients with Incurable Lung Cancer. J. Palliat. Med..

[58] McDonald J., Swami N., Pope A., Hales S., Nissim R., Rodin G., Hannon B., Zimmermann C. (2018). Caregiver Quality of Life in Advanced Cancer: Qualitative Results from a Trial of Early Palliative Care. Palliat. Med..

[59] Bigi S., Ganfi V., Borelli E., Potenza L., Artioli F., Eliardo S., Mucciarini C., Cottafavi L., Cruciani M., Cacciari C. (2022). Perceptions of Hope among Bereaved Caregivers of Cancer Patients Who Received Early Palliative Care: A Content and Lexicographic Analysis. Oncologist.

[60] Koch A., Mantzouris S. (2020). Nurses’ Role in Providing Comprehensive Communication, Prognostication, and Palliative Care During the COVID-19 Pandemic. J. Hosp. Palliat. Nurs. JHPN Off. J. Hosp. Palliat. Nurses Assoc..

[61] Faria C., Branco V., Ferreira P., Gouveia C., Trevas S. (2021). Total Pain Management and a Malignant Wound: The Importance of Early Palliative Care Referral. Cureus.

[62] Borelli E., Bigi S., Potenza L., Gilioli F., Artioli F., Porzio G., Porro C.A., Efficace F., Bruera E., Luppi M. (2022). Gratitude among Advanced Cancer Patients and Their Caregivers: The Role of Early Palliative Care. Front. Oncol..

[63] Bigi S., Ganfi V., Borelli E., Potenza L., Artioli F., Eliardo S., Mucciarini C., Cottafavi L., Ferrari U., Lombardo L. (2023). Perceptions of Death among Patients with Advanced Cancer Receiving Early Palliative Care and Their Caregivers: Results from a Mixed-Method Analysis. Oncologist.

[64] White N., Packard K., Kalkowski J., Bradley T. (2021). Building Hopefulness Through Financial Education and Coaching. Am. J. Lifestyle Med..

[65] Bravin A.M., Trettene A.D.S., Andrade L.G.M.D., Popim R.C. (2019). Benefits of Spirituality and/or Religiosity in Patients with Chronic Kidney Disease: An Integrative Review. Rev. Bras. Enferm..

[66] Saleh Manijeh H., Rostami M., Ahmadboukani S. (2021). Development of the Coronavirus Anxiety Model in the Elderly: Based on Hope and Health-Related Quality of Life With the Mediating Role of Perceived Social Support. Gerontol. Geriatr. Med..

[67] Cohen M.G., Althouse A.D., Arnold R.M., Bulls H.W., White D.B., Chu E., Rosenzweig M.Q., Smith K.J., Schenker Y. (2022). Hope and Advance Care Planning in Advanced Cancer: Is There a Relationship?. Cancer.

[68] DeMartini J., Fenton J.J., Epstein R., Duberstein P., Cipri C., Tancredi D., Xing G., Kaesberg P., Kravitz R.L. (2019). Patients’ Hopes for Advanced Cancer Treatment. J. Pain Symptom Manag..

[69] Altınışık M., Kocabıyık B., Arıkan F., Şevik H.Y., Coşkun H.Ş. (2022). The Relationship between Hope Levels and Unmet Needs of Caregivers of Advanced Cancer Patients†. Jpn. J. Nurs. Sci..

[70] Lohne V., Miaskowski C., Rustøen T. (2012). The Relationship Between Hope and Caregiver Strain in Family Caregivers of Patients With Advanced Cancer. Cancer Nurs..

[71] Duggleby W., Wright K., Williams A., Degner L., Cammer A., Holtslander L. (2007). Developing a Living with Hope Program for Caregivers of Family Members with Advanced Cancer. J. Palliat. Care.

[72] Rumpold T., Schur S., Amering M., Ebert-Vogel A., Kirchheiner K., Masel E., Watzke H., Schrank B. (2017). Hope as Determinant for Psychiatric Morbidity in Family Caregivers of Advanced Cancer Patients: Hope as Determinant for Psychiatric Morbidity in Family Caregivers. Psychooncology.

[73] Odejide O.O. (2020). Strategies for Introducing Palliative Care in the Management of Relapsed or Refractory Aggressive Lymphomas. Hematology.

[74] Odejide O.O., Cronin A.M., Condron N.B., Fletcher S.A., Earle C.C., Tulsky J.A., Abel G.A. (2016). Barriers to Quality End-of-Life Care for Patients With Blood Cancers. J. Clin. Oncol..

[75] Bandieri E., Borelli E., Gilioli F., Bigi S., Mucciarini C., Ferrari U., Eliardo S., Pinto L., Porro C.A., Efficace F. (2023). Stigma of Palliative Care among Patients with Advanced Cancer and Their Caregivers on Early Palliative Care. Cancers.

[76] Snyder C.R. (1994). The Psychology of Hope: You Can Get There from Here.

[77] Levy M.H., Adolph M.D., Back A., Block S., Codada S.N., Dalal S., Deshields T.L., Dexter E., Dy S.M., Knight S.J. (2012). Palliative Care. J. Natl. Compr. Cancer Netw. JNCCN.

[78] Clark D. (1999). `Total Pain’, Disciplinary Power and the Body in the Work of Cicely Saunders, 1958–1967. Soc. Sci. Med..

[79] Walling A., Lorenz K.A., Dy S.M., Naeim A., Sanati H., Asch S.M., Wenger N.S. (2008). Evidence-Based Recommendations for Information and Care Planning in Cancer Care. J. Clin. Oncol. Off. J. Am. Soc. Clin. Oncol..

[80] Mack J.W., Wolfe J., Grier H.E., Cleary P.D., Weeks J.C. (2006). Communication About Prognosis Between Parents and Physicians of Children With Cancer: Parent Preferences and the Impact of Prognostic Information. J. Clin. Oncol..

[81] Lelorain S., Brédart A., Dolbeault S., Sultan S. (2012). A Systematic Review of the Associations between Empathy Measures and Patient Outcomes in Cancer Care. Psychooncology.

[82] Nipp R.D., Greer J.A., El-Jawahri A., Moran S.M., Traeger L., Jacobs J.M., Jacobsen J.C., Gallagher E.R., Park E.R., Ryan D.P. (2017). Coping and Prognostic Awareness in Patients With Advanced Cancer. J. Clin. Oncol..

[83] Greer J.A., Jacobs J.M., El-Jawahri A., Nipp R.D., Gallagher E.R., Pirl W.F., Park E.R., Muzikansky A., Jacobsen J.C., Jackson V.A. (2018). Role of Patient Coping Strategies in Understanding the Effects of Early Palliative Care on Quality of Life and Mood. J. Clin. Oncol..

[84] Borelli E., Bigi S., Potenza L., Eliardo S., Artioli F., Mucciarini C., Cottafavi L., Cagossi K., Razzini G., Cruciani M. (2021). Changes in Cancer Patients’ and Caregivers’ Disease Perceptions While Receiving Early Palliative Care: A Qualitative and Quantitative Analysis. Oncologist.

[85] Wood A.M., Froh J.J., Geraghty A.W.A. (2010). Gratitude and Well-Being: A Review and Theoretical Integration. Clin. Psychol. Rev..

[86] Wood A.M., Maltby J., Gillett R., Linley P.A., Joseph S. (2008). The Role of Gratitude in the Development of Social Support, Stress, and Depression: Two Longitudinal Studies. J. Res. Personal..

[87] Althaus B., Borasio G.D., Bernard M. (2018). Gratitude at the End of Life: A Promising Lead for Palliative Care. J. Palliat. Med..

[88] Centeno C., Arantzamendi M., Rodríguez B., Tavares M. (2010). Letters from Relatives: A Source of Information Providing Rich Insight into the Experience of the Family in Palliative Care. J. Palliat. Care.

[89] Back A.L. (2020). Patient-Clinician Communication Issues in Palliative Care for Patients With Advanced Cancer. J. Clin. Oncol..

